# Supplementary material for: Modeling glioblastoma heterogeneity as a dynamic network of cell states
Source: Mol Syst Biol. 2021 Sep 16;17(9):e10105. doi: 10.15252/msb.202010105 (PMC8444284; doi:10.15252/msb.202010105)
Supplement: Supplementary file 6 — Source Data for Figure 5 [file MSB-17-e10105-s004.zip › Figure5A_sourcedata/GSEA_3071/hallmarks_stateI.GseaPreranked.1623742531165/HALLMARK_IL2_STAT5_SIGNALING.html]

Details for gene set HALLMARK\_IL2\_STAT5\_SIGNALING[GSEA]

|  || Dataset | state13071 |
| Phenotype | NoPhenotypeAvailable |
| Upregulated in class | na\_pos |
| GeneSet | HALLMARK\_IL2\_STAT5\_SIGNALING |
| Enrichment Score (ES) | 0.48205316 |
| Normalized Enrichment Score (NES) | 2.610938 |
| Nominal p-value | 0.0 |
| FDR q-value | 0.0 |
| FWER p-Value | 0.0 |
Table: GSEA Results Summary

  

Fig 1: Enrichment plot: HALLMARK\_IL2\_STAT5\_SIGNALING      
 Profile of the Running ES Score & Positions of GeneSet Members on the Rank Ordered List

  

| PROBE | GENE SYMBOL | GENE\_TITLE | RANK IN GENE LIST | RANK METRIC SCORE | RUNNING ES | CORE ENRICHMENT || 1 | SPP1 |  |  | 0 | 2.808 | 0.0669 | Yes |
| 2 | PTCH1 |  |  | 7 | 1.951 | 0.1116 | Yes |
| 3 | COL6A1 |  |  | 10 | 1.797 | 0.1538 | Yes |
| 4 | ITIH5 |  |  | 35 | 1.473 | 0.1819 | Yes |
| 5 | SCN9A |  |  | 46 | 1.381 | 0.2119 | Yes |
| 6 | NRP1 |  |  | 81 | 1.180 | 0.2302 | Yes |
| 7 | F2RL2 |  |  | 87 | 1.137 | 0.2558 | Yes |
| 8 | PLEC |  |  | 101 | 1.085 | 0.2779 | Yes |
| 9 | SLC2A3 |  |  | 109 | 1.060 | 0.3011 | Yes |
| 10 | ITGAV |  |  | 117 | 1.029 | 0.3236 | Yes |
| 11 | EMP1 |  |  | 122 | 1.012 | 0.3465 | Yes |
| 12 | AHNAK |  |  | 163 | 0.887 | 0.3560 | Yes |
| 13 | ANXA4 |  |  | 190 | 0.847 | 0.3687 | Yes |
| 14 | SLC1A5 |  |  | 199 | 0.831 | 0.3861 | Yes |
| 15 | XBP1 |  |  | 215 | 0.797 | 0.4008 | Yes |
| 16 | IFITM3 |  |  | 268 | 0.714 | 0.4027 | Yes |
| 17 | P4HA1 |  |  | 291 | 0.688 | 0.4127 | Yes |
| 18 | SOCS1 |  |  | 292 | 0.686 | 0.4290 | Yes |
| 19 | TIAM1 |  |  | 341 | 0.636 | 0.4302 | Yes |
| 20 | WLS |  |  | 360 | 0.625 | 0.4399 | Yes |
| 21 | TNFSF10 |  |  | 395 | 0.590 | 0.4441 | Yes |
| 22 | CKAP4 |  |  | 405 | 0.582 | 0.4553 | Yes |
| 23 | IFNGR1 |  |  | 407 | 0.580 | 0.4689 | Yes |
| 24 | ENPP1 |  |  | 410 | 0.578 | 0.4821 | Yes |
| 25 | NCOA3 |  |  | 514 | 0.499 | 0.4640 | No |
| 26 | GABARAPL1 |  |  | 544 | 0.484 | 0.4671 | No |
| 27 | RHOB |  |  | 609 | 0.447 | 0.4592 | No |
| 28 | KLF6 |  |  | 647 | 0.433 | 0.4588 | No |
| 29 | FURIN |  |  | 663 | 0.426 | 0.4646 | No |
| 30 | BHLHE40 |  |  | 720 | 0.405 | 0.4580 | No |
| 31 | TWSG1 |  |  | 747 | 0.398 | 0.4599 | No |
| 32 | GUCY1B1 |  |  | 887 | 0.353 | 0.4280 | No |
| 33 | SNX9 |  |  | 909 | 0.348 | 0.4302 | No |
| 34 | CD81 |  |  | 1059 | 0.315 | 0.3945 | No |
| 35 | PLAGL1 |  |  | 1132 | 0.301 | 0.3807 | No |
| 36 | MXD1 |  |  | 1151 | 0.298 | 0.3826 | No |
| 37 | ARL4A |  |  | 1195 | 0.289 | 0.3770 | No |
| 38 | CAPG |  |  | 1222 | 0.285 | 0.3763 | No |
| 39 | PHLDA1 |  |  | 1262 | 0.275 | 0.3715 | No |
| 40 | PLPP1 |  |  | 1395 | 0.252 | 0.3392 | No |
| 41 | CD44 |  |  | 1430 | -0.251 | 0.3353 | No |
| 42 | TGM2 |  |  | 1444 | -0.252 | 0.3375 | No |
| 43 | CCND3 |  |  | 1482 | -0.255 | 0.3329 | No |
| 44 | CDKN1C |  |  | 1571 | -0.264 | 0.3136 | No |
| 45 | CASP3 |  |  | 1639 | -0.273 | 0.3007 | No |
| 46 | NCS1 |  |  | 1647 | -0.273 | 0.3052 | No |
| 47 | SYNGR2 |  |  | 1939 | -0.312 | 0.2281 | No |
| 48 | BCL2L1 |  |  | 1954 | -0.313 | 0.2315 | No |
| 49 | POU2F1 |  |  | 2010 | -0.318 | 0.2232 | No |
| 50 | APLP1 |  |  | 2066 | -0.328 | 0.2150 | No |
| 51 | BATF3 |  |  | 2070 | -0.329 | 0.2220 | No |
| 52 | CCNE1 |  |  | 2087 | -0.332 | 0.2252 | No |
| 53 | AHCY |  |  | 2089 | -0.333 | 0.2329 | No |
| 54 | DCPS |  |  | 2194 | -0.351 | 0.2110 | No |
| 55 | PNP |  |  | 2223 | -0.358 | 0.2114 | No |
| 56 | ETV4 |  |  | 2325 | -0.378 | 0.1911 | No |
| 57 | UMPS |  |  | 2368 | -0.387 | 0.1881 | No |
| 58 | ITGA6 |  |  | 2372 | -0.388 | 0.1965 | No |
| 59 | BMP2 |  |  | 2589 | -0.436 | 0.1442 | No |
| 60 | SPRY4 |  |  | 2916 | -0.552 | 0.0628 | No |
| 61 | TNFRSF21 |  |  | 3004 | -0.604 | 0.0519 | No |
| 62 | GPX4 |  |  | 3103 | -0.679 | 0.0396 | No |
| 63 | RGS16 |  |  | 3134 | -0.708 | 0.0478 | No |
| 64 | CDC6 |  |  | 3204 | -0.785 | 0.0465 | No |
| 65 | ODC1 |  |  | 3257 | -0.860 | 0.0519 | No |
| 66 | SYT11 |  |  | 3286 | -0.904 | 0.0653 | No |
Table: GSEA details [plain text format]

  

Fig 2: HALLMARK\_IL2\_STAT5\_SIGNALING: Random ES distribution      
 Gene set null distribution of ES for **HALLMARK\_IL2\_STAT5\_SIGNALING**
